# Probabilistic Prognostic Estimates of Survival in Metastatic Cancer Patients (PPES-Met) Utilizing Free-Text Clinical Narratives

**DOI:** 10.1038/s41598-018-27946-5

**Published:** 2018-07-03

**Authors:** Imon Banerjee, Michael Francis Gensheimer, Douglas J. Wood, Solomon Henry, Sonya Aggarwal, Daniel T. Chang, Daniel L. Rubin

**Affiliations:** 1 Department of Biomedical Data Science, Stanford University, Stanford, CA USA; 2 Department of Radiation Oncology, Stanford University, Stanford, CA USA; 3 Biomedical Data Science, Radiology, and Medicine (BMIR) Stanford University, Stanford, CA USA

## Abstract

We propose a deep learning model - Probabilistic Prognostic Estimates of Survival in Metastatic Cancer Patients (PPES-Met) for estimating short-term life expectancy (>3 months) of the patients by analyzing free-text clinical notes in the electronic medical record, while maintaining the temporal visit sequence. In a single framework, we integrated semantic data mapping and neural embedding technique to produce a text processing method that extracts relevant information from heterogeneous types of clinical notes in an unsupervised manner, and we designed a recurrent neural network to model the temporal dependency of the patient visits. The model was trained on a large dataset (10,293 patients) and validated on a separated dataset (1818 patients). Our method achieved an area under the ROC curve (AUC) of 0.89. To provide explain-ability, we developed an interactive graphical tool that may improve physician understanding of the basis for the model’s predictions. The high accuracy and explain-ability of the PPES-Met model may enable our model to be used as a decision support tool to personalize metastatic cancer treatment and provide valuable assistance to the physicians.

## Introduction

In the United States, around 500,000 patients develop metastatic cancer every year^1^. Optimal treatment decisions for metastatic cancer are often not clear, and there is variation in clinical practice. Several clinical studies^2,3^ have shown over-utilization of aggressive medical interventions and protracted radiation treatment courses for terminally ill patient with metastatic cancer. According to the reporting in numerous scientific reviews^4,5^, this is mainly due to that fact that physicians are overly-optimistic during survival prediction of patients with terminal cancer. Earlier studies^6,7^ suggested that the radiation oncologists’ predicted survival for patients with a short life span (3 months) is approximately double than the actual survival. This is also supported by an early stage results of an ongoing prospective study on 899 patients enrolled in a palliative radiation study conducted by the Stanford Radiation Oncology department. The study demonstrates that only 62% oncologists’ estimations of short-term life expectancy (0–3 months) had actual survival falling within this time interval, which is inadequate to conduct precise planning of personalized palliative cancer treatment. This may lead physicians to choose overly-aggressive treatments for some patients, with increased side effects and costly health care bills, while other patients may be under-treated and denied access to effective treatments that could reduce symptoms or even extend survival.

With the advancement of AI, prediction of disease trajectory using electronic medical records (EMR) has gained a significant interest from the bioinformatics community^8–12^. However, the irregular time gaps between the clinical events have not been adequately modeled in the existing studies. This mainly due to the fact that traditional ML models often use simple sequential pattern mining solutions to identify complex temporal phenotype^13^. The Markovian models^14,15^ being memoryless, are also insufficient to model long-term event dependencies. Therefore, a patient who has a routine checkup with some affirmative medical statement (e.g., “currently doing well” at *t*_*n*+1_) would destroy the illness history (e.g. “bleeding increased” at *t*_*n*_). This aspect is particularly problematic for terminal conditions (e.g. metastatic cancer) where the events are irregular and should be weighted based on persisting temporal context.

Deep learning is currently a promising way to make sense of a large volume of medical data. Several recent works^11,16,17^ employ deep learning approach to EMR for predicting future events. DoctorAI^18^ uses an RNN model for predicting medical events and time gap based on structural information - ICD-9 codes, medication codes, and procedure codes. DeepCare^19^ creates an interesting analogy between natural languages and structural medical records, and uses a dynamic memory model for predicting medical outcomes. Deepr^16^ employs convolutional neural net on the structural medical records (ICD9 codes) for predicting future risk. However, ICD9 or the CPT codes are often not reliable due to the error-prone manual assignment process and bias related to use of these codes for billing. Studies have found the error rates may be up to 70 percent^20^. Lipton *et al*.^21^ used pre-selected 13 vitals, formatted as structured variables, as input to a deep learning model to predict diagnosis.

On the other hand, clinical narratives are unique rich components of that the medical record that contain crucial details about patient status and expert insights in a descriptive form, and they could have a significant impact on predictive performance. However, prior work to date has largely excluded free-text narratives likely because they pose complex challenges, including unstructured representation, high dimensionality, and sparsity. Natural language processing (NLP) tools are designed to encode unstructured text into computer manageable representation. However, most existing predictive models are either limited using solely structural data (lab values, demographics)^10,12^ or have adopted relatively simplistic information extraction from unstructured narratives (bag of words (BoW) and term frequency-inverse document frequency)^11^. Such sparse approaches for information extraction from clinical narratives face several challenges in the clinical domain: (i) scalability - BoW encode every word in the vocabulary as one-hot-encoded vector, but clinical vocabulary may potentially run into millions; (ii) semantics of the words - the vectors corresponding to same contextual words are orthogonal; (iii) word orderings - BoW models also don’t consider the order of words in the phrase. Kwong *et al*.^22^ recently proposed a system for detection of atrial fibrillation (AF) in post-cryptogenic stroke (CS) or transient ischemic attack (TIA) using a combination of clinical notes and structured EHR data. For representing the unstructured notes, authors extract only positive mentions of disease concepts from the clinical notes which is a restricted representation and may not be applicable to the metastatic cancer domain where not only disease, but various contexts of the notes could be important, including performance status, imaging findings, tolerance to systemic therapy.

An emerging recent trend in deep learning with text is to adopt a distributed representation of word meaning by constructing a “neural embedding” of each word or document. The Word2Vec model introduced by Mikolov *et al*.^23^ is the most popular approach for providing semantic word embeddings. One of the biggest challenges with word2vec, however, is handling unknown or out-of-vocabulary (OOV) words and morphologically similar words (abbreviations, acronyms, telegraphic phases). This can particularly be an issue in medicine where synonyms and related words can be used depending on the preferred style of radiologist, and words may be used infrequently in a large corpus. If the word2vec model has not encountered a word before, it will be forced to use a random vector, which is generally far from its ideal representation.

In addition to the foregoing challenges, deep learning/AI models for clinical outcome prediction are limited in their ability to provide explanation of the basis for their predictions; their nested non-linear structure make them highly non-transparent and work as a ‘black-box’. We refer to this limitation as “explain-ability”. The need for explain-ability is particularly important in clinical prediction where heterogeneous data from multiple sources have been incorporated in a single model. Recently, development of advanced techniques for “peering inside the deep learning black box” models have gained attention, particularly development of methods that help to better understand what the deep learning model has learned for the object identification task from images^24,25^ as well as techniques for explaining individual predictions^26,27^. For natural language classification tasks, sensitivity analysis methods^28^ have been developed to visualize the impact of input words on the output decision of the deep learning models, which takes the partial derivative of the loss function with respect to each input variable. However, limited research effort has been pursued towards explain-ability of clinical predictive analysis with sequence-dependent EMR data. Without a formalized mechanism to reason about why the computerized model predicts a specific outcome at a particular timepoint, clinicians tend to distrust them, which limits the validation and adoption of highly efficient and technologically advanced AI-models into clinical practice.

**Figure 1:**
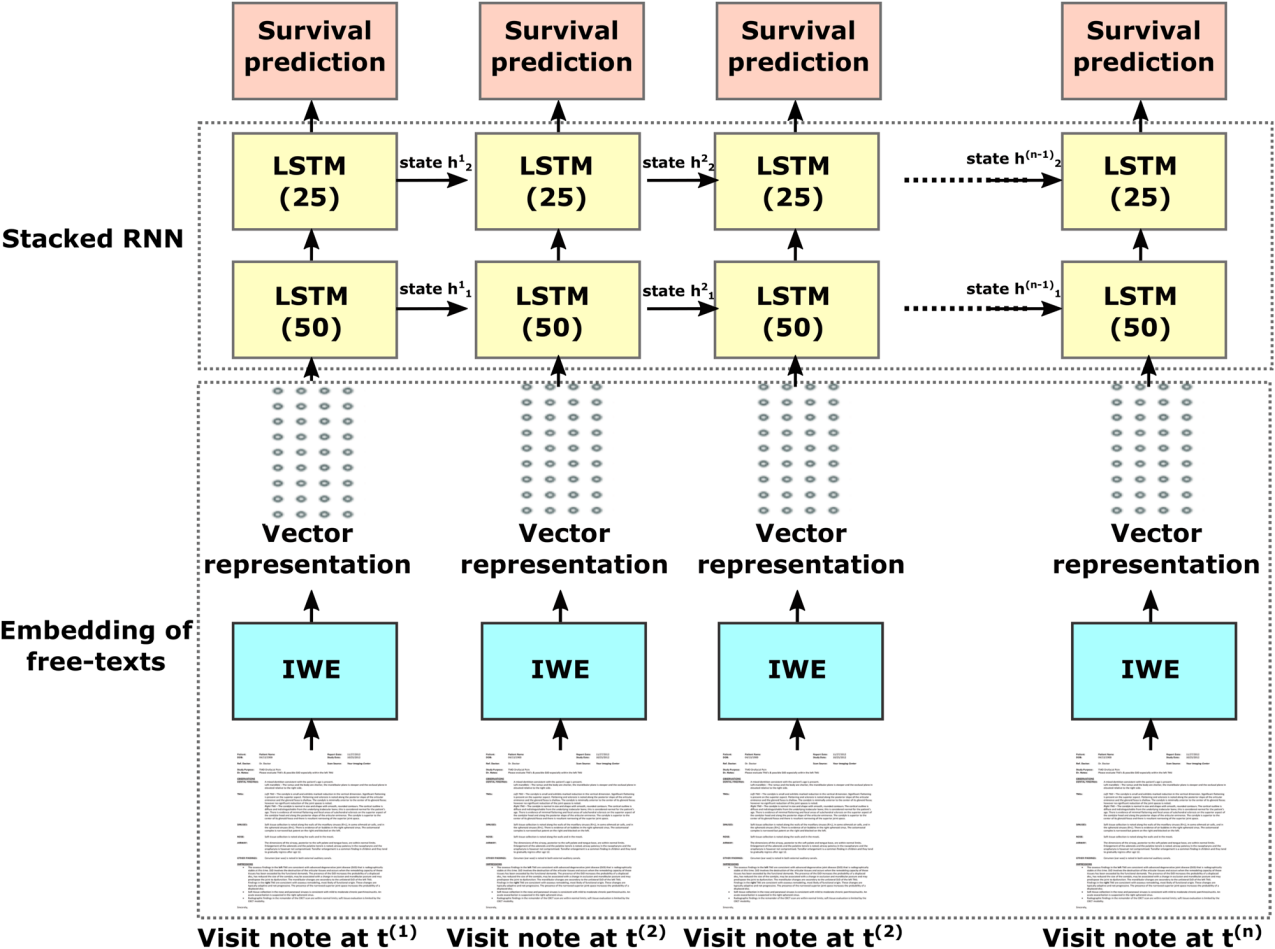
Workflow of the proposed system - PPES-Met. IWE = intelligent word embedding. LSTM = long short term memory. Number in parentheses indicates dimension of input vector to LSTM.

The Stanford Cancer Institute Research Database (SCIRDB) holds electronic clinic note data for over 20,000 patients with metastatic cancer. Using this comprehensive dataset, we created a dynamic deep learning model - Probabilistic Prognostic Estimates of Survival in Metastatic Cancer Patients (PPES-Met). Figure 1 presents an overview of the proposed pipeline comprising our system, which takes as input a sequence of clinical narratives (e.g. radiology reports, oncologist notes, discharge summaries) ordered according to the date of visits, and computes as output a probability of short-term life expectancy (>3 months) for each visit considering the current and all the historic time point.

The complexity of the model mainly lies in extracting relevant information from the heterogeneous types of free-text clinical notes along with modeling temporal irregularity of the visits. The proposed model also generates an interactive patient-level summary of the predictions that enables physicians to view the events that trigger the predictions to provide explanation. The PPES-Met model was validated on a combination of a general group of metastatic patients and 899 patients enrolled in a prospective survey study of providers’ ability to estimate prognosis of patients receiving palliative radiation therapy.

The main research contributions of this paper can be summarized as follows:

1. Developed a hybrid pipeline that combines semantic data mining with neural embedding for creating context-aware dense vector representation of the multiple types of free-text clinical notes.
2. Proposed an efficient deep prognosis model that takes as input the context-aware vectorized representation of sequential clinic notes and outputs a probability of short-term life expectancy estimate (>3 months).
3. Incorporated an interactive visualization method for improving physician understanding of the basis for the model’s predictions.

## Experimental Setup

With the approval of the Stanford University Institutional Review Board (IRB), we created a “Metastatic cancer database” (MetDB) which includes adult cancer patients (13,523) seen at the Stanford Cancer Center from 2008–2017 and diagnosed with distant metastases (see Table 1). We confirm that all experiments presented in the study were performed in accordance with relevant guidelines and regulations of IRB, and informed consent was obtained from all participants and/or their legal guardians. The two core inclusion criteria of the patients are – (i) if the patient is in the Stanford Cancer Registry and has at least a cancer stage M1. This stage information is considered to be accurate since this is audited by humans; (ii) if the patient has two separate procedures with ICD9 diagnosis codes to indicate metastatic disease. For instance, 198.5 is “secondary malignant neoplasm of bone and bone marrow” and is a reliable indicator of the patient having bone metastasis. These two inclusion criteria cut down the false positives dramatically. We also audited a random selection (~50) of patients from MetDB and found that almost all of them had distant metastasis.

**Table 1:** Characteristics of complete dataset, including analyzed and non-analyzed patients.

Characteristic	Metastatic cancer database (MetDB)	Palliative radiation dataset (PrDB)
No. of patients	13,523	899
Age	61.5 (IQR 51.2–70.5)	65.0 (IQR 55.8–72.2)
Sex	M: 6621 (49%); F: 6902 (51%)	M: 460 (51.1%); F: 439 (48.9%)
*Primary site	Breast: 1493 (11.0%)	Breast: 141 (15.7%)
	Endocrine: 211 (1.6%)	Endocrine: 0 (0%)
	Gastrointestinal: 3575 (26.4%)	Gastrointestinal: 145 (16.1%)
	Genitourinary: 1504 (11.1%)	Genitourinary: 112 (12.5%)
	Gynecologic: 849 (6.3%)	Gynecologic: 50 (5.6%)
	Head and neck: 506 (3.7%)	Head and neck: 57 (6.3%)
	Skin: 453 (3.3%)	Skin: 122 (13.6%)
	Thorax: 2178 (16.1%)	Thorax: 252 (28.0%)
	Other/Multiple/Unknown: 2754(20.4%)	Other/Multiple/Unknown: 20 (2.2%)
Note types	Oncology notes, inpatient notes, radiology notes and reports, lab reports, treatment notes, operative reports	

This database contains various types of free-text visit notes (e.g. oncologist notes, inpatient notes, ICU notes) from date of metastatic cancer diagnosis to death. A separate database (“Palliative radiation dataset” (PrDB)) was obtained using patients (899) enrolled from 2015–2016 in a prospective survey study conducted in our institution’s Radiation Oncology department(see Table 1). In the PrDB, all the patients who were to receive palliative radiation therapy (any treatment for incurable metastatic cancer) were included. Characteristics of both datasets are listed in Table 1.

We excluded 1,499 patients from MetDB due to a lack of follow-up information. Of the remaining 12,024 patients, 7,475 (62%) patients have died. MetDB patients and PrDB patients were seen in the Stanford Health Care system for 471,005 daily encounters/visits, including outpatient and inpatient contact (each day of a hospitalization was counted as a separate visit). For these visits, median follow-up was 12.7 months. Median overall survival was 22.4 months. Patients were hospitalized for 115,716 (24.6%) visits. There were 1,403,544 provider notes. The 12,024 analyzed MetDB patients and 899 PrDB patients were randomly divided into a training and validation set of 10,239 patients with 380,080 visits, validation set of 1,785 patients and test set of 1,818 patients (15%) with 90,925 visits. All the PrDB patients were placed only in the test set.

**Figure 2:**
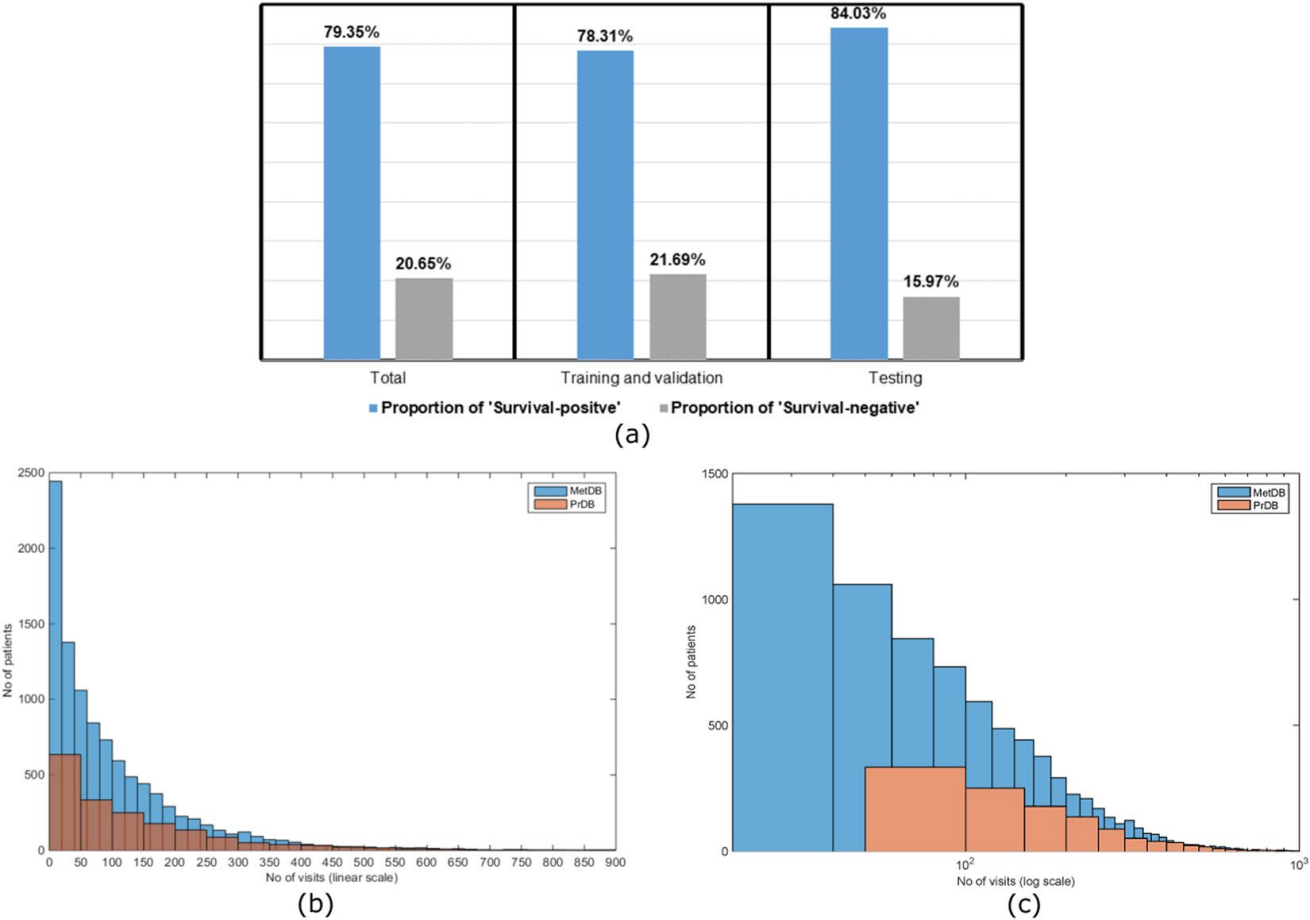
Statistics of the dataset. (**a**) Sample distribution in the cohort. Distribution of visits in the cohort: linear scale (**b**) and logarithmic scale (**c**); Patients with less than 2 visits were not included in both MetDB and PrDB.

We trained the PPES-Met model (Fig. 1) on the training set of 10,293 patients, validated on 1,785 patients, and tested the performance on 1818 patients included in the test set. As ground truth label, we used 3-month survival record defined at each time point, and converted to categorical class labels for probabilistic prediction where category 1: “Survival - positive” stands for survival past 3 months starting from the current visit date; category 2: “Survival - negative” flagged the non-survival. In Fig. 2a, we present the distribution of “Survival - positive” and “Survival - negative” samples in our complete cohort along with individual distribution in training, validation, and test datasets.

In both MetDB and PrDB, there is a wide variation in the total number of individual visits (see Fig. 2b [linear scale] and c.[log scale]). In fact, a very few patients have more than 1000 visits in both training and testing dataset. Therefore, we pad each input sequences with zeros when the sequence is shorter than 1000 and truncated the historic visits when sequence is longer than 1000. The zero pad doesn’t affect the outcome, since the real visit data never contain only zero values which makes it trivial for the model to disambiguate between true and padded data. The model was trained with 0.001 learning rate with 0.0001 decay/epoch and random shuffling was applied in the batch. We adopted a weighted cross entropy loss function (see Methods) with 2x weight to survival and 1x weight to the non-survival data points, and 0.1x weight to the padded data point to normalize the learning accuracy.

## Results

In order to evaluate the performance of the PPES-Met model in a robust way, we follow a dual evaluation strategy – (i) quantitative evaluation: measure the overall prognosis estimation accuracy using the standard metrics; (ii) qualitative evaluation: evaluate the patient-level performance and perform error analysis with intelligible longitudinal graph summary for understanding the basis of prediction.

### Quantitative evaluation

**Figure 3:**
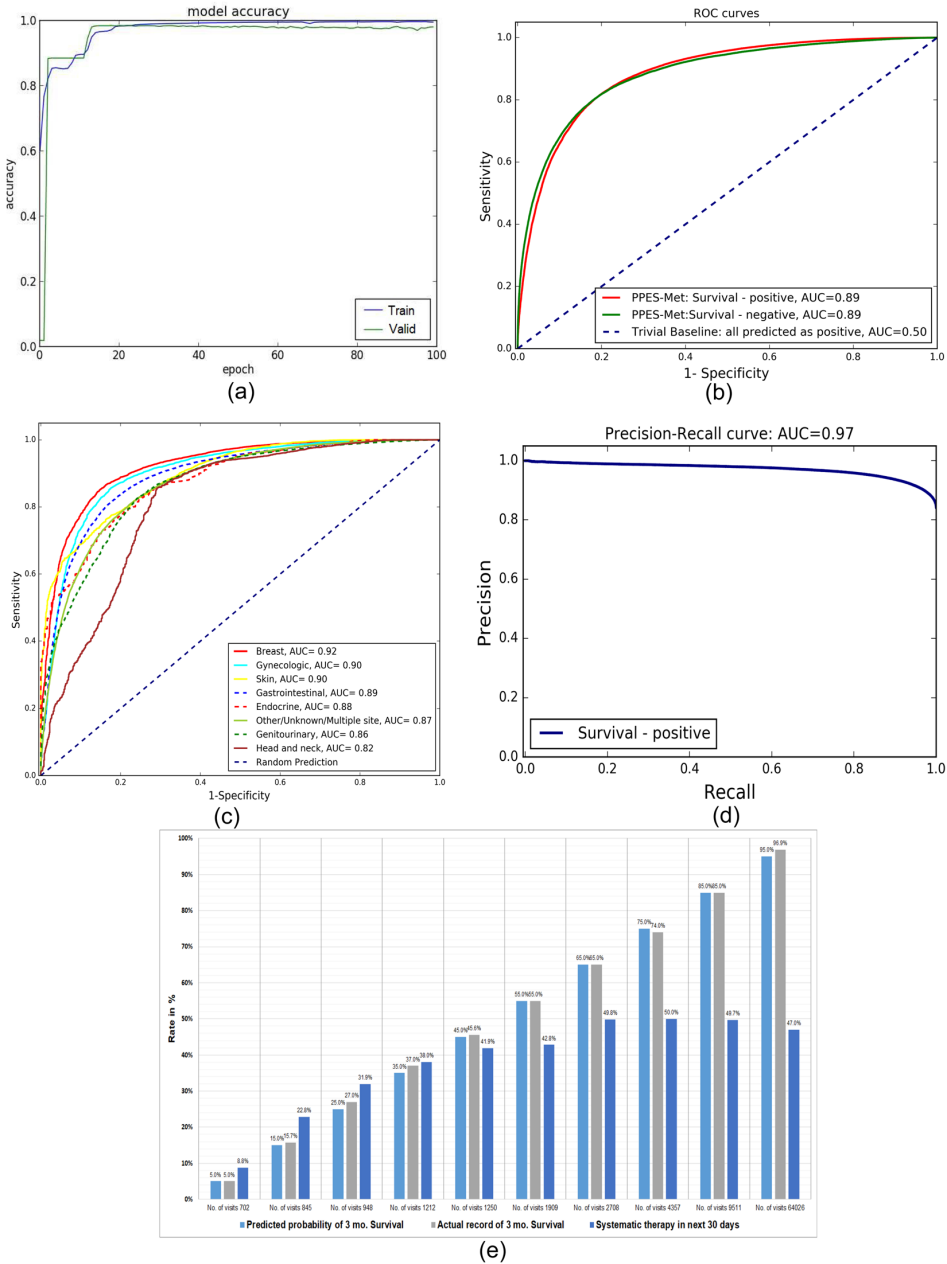
Overall quantitative performance. (**a**) Epoch based performance accuracy. (**b**) ROC curve on the test set without padding values. (**c**) ROC curves on the test set separated based on the primary site. (**d**) Precision-Recall curve on the test set without padding values. (**e**) Correlation between predicted probability and actual survival rates, compared with systematic therapy information in next 30 days shows that many patients are getting aggressive therapy when the rate of survival is limited while the rate of systemic therapy utilization plateaus at around 50% for patients with high expected survival.

In Fig. 3a, we present the performance of model on the training and the validation set measured during training phase as mean accuracy rate across both “Survival - positive” and “Survival - negative” labels. After 100 epochs, the validation accuracy was about 0.98 and the training accuracy is about 0.998. The high accuracy on the validation set is an indication that the model is not over-fitted to the training set. As seen from the figure, the model was mostly saturated after 30 epochs. However, the accuracy value is over-optimistic due to inclusion of padding values.

To evaluate the actual performance of survival estimation on the test dataset without padding values, we adopted the Receiver Operating Characteristic (ROC) curve as primary metric. ROC curve is a well-accepted method to show the trade-off between true-positive and false-positive where the models produced a sort of scores for test samples, and presents pairs of specificity and sensitivity values calculated at all possible threshold scores. The ROC curve also provides a single performance measure called the Area under the ROC curve (AUC) score where AUC 1 represents a perfect test and 0.5 represents a worthless test. The ROC curve for PPES-Met prediction on the testset of both “Survival - positive”: >3 months survival and “Survival - negative”: <3 months survival is shown in Fig. 3b where x-axis represents 1- specificity, y-axis represents sensitivity, and the curve covers all possible thresholds (cut-off points). We are validating PPES-Met’s prediction against an imbalanced test set where 80% of the datapoints are in “Survival positive” catergory (see Fig. 2), thus we compare the performance of our model against a trivial baseline which will always predict that a patient will survive 3 months as it will have 80% prediction accuracy. However, the AUC score for the trivial baseline is 0.50 (same as the random prediction) where as our model scored 0.89 since AUC is irrespective of the actual positive/negative balance in the test set. The best cut-off that maximizes (sensitivity + specificity) is observed to be 0.83 for “Survival - positive” category. The resultant AUC is 0.89 with 95% confidence interval [0.884–0.897] which reflects consistent high survival prediction accuracy of our model. Note that the overall accuracy is estimated by comparing the true survival data with prediction of each individual entry in the time-to-event table. Therefore, if a patient’s condition was evaluated four times in a day (e.g. critical care team – nurse – radiologist – oncologist), that patient would contribute four entries in the time-to-event table.

In order to check the influence of primary site on the prediction, we calculated the AUC separately for each primary site in Fig. 3c. As seen from the figure that the AUC ranges from 0.92 (Breast) to 0.82 (Head and neck), and AUCs from most of the primary sites are >=0.86. Greater than 0.82 AUC shows that our model is performing equally well for all the primary sites irrespective of the linguistic variation in the clinical note content. To check the calibration with the ground truth, we also measure the Brier score which computes the mean squared difference between the predicted probability assigned and the actual outcome of the survival and the value ranged between 0 and 1. Therefore, the lower the Brier score is for a set of predictions, the better the predictions are calibrated. The Brier score for PPES-Met model survival prediction was 0.069 which shows the prediction was highly calibrated with the ground truth. In Fig. 3d, we presented the third evaluation metric - Precision-Recall curve for PPES-Met model where our model model achieved a high AUC value (0.97). This shows that PPES-Met prediction achieved a high precision as well as high recall on the test set (see the Supplementary materials for more evaluation).

In addition to the standard statistical metrics, in Fig. 3e, we plotted rate of predicted chance of survival (in %) against actual survival rate of the patients as a bar plot for providing a more intutive quantitative representation of our prediction performance. In order to compute rate of predicted chance of survival (in %), we binned the predicted probability in decile in such a way that the 1^*st*^ bin contains the visits with 0–10% predicted probability of survival, the 2^*nd*^ bin contains the visits with 10.1–20% predicted probability of survival, and so on. In the bottom of each bar, we mentioned the number of sample visit in each bin. The bar plot shows that prediction by our model and actual survival rate has a very good correlation. For instance, in the sub-population, only 5% patients survived when the PPES-Met’s average predicted rate of survival is 5%, while when PPES-Met’s predicted survival probability is 95%, 96.9% patients survived.

### Qualitative evaluation

The overall high accuracy of the sequence-dependent PPES-Met model measured by multiple statistical metrics (see Fig. 3) suggests that the proposed model performed quite well on estimating short-term survival (>3 months) for the validation and test set. However, without a formalized mechanism to reason about why the computerized model predicts a specific outcome at a particular timepoint, clinicians tend to doubt the prediction which limits the adaptability of the model. Therefore, for deriving human interpretable explanation of PPES-Met survival prediction, we implemented an interactive graphical interface that generates a longitudinal probabilistic summary for each patient by exploiting the trained two-stage PPES-Met model. In Fig. 3, we present the graph summary for multiple randomly selected patients which shows that predicted probability sequence closely follows the ground truth labels.

**Figure 4:**
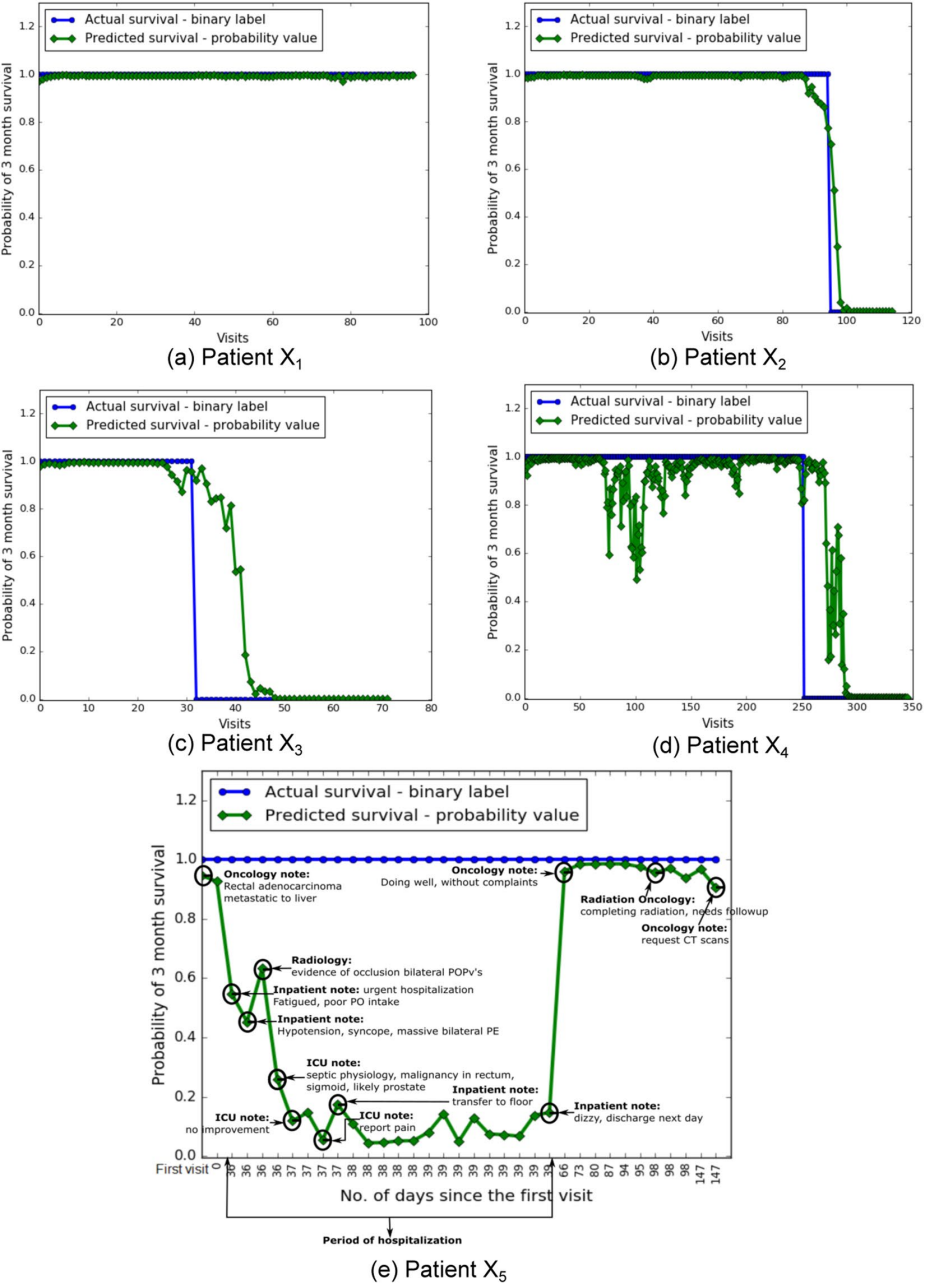
Patient-level prognosis estimate – x-axis shows the visit index in ascending order starting from the first visit, y-axis shows the probability of survival. Blue line represents the ground truth: Survival - positive = 1, Survival - negative = 0. Green line represents the predicted survival probability score: in (**a** and **b**) predicted probability sequence follows the actual survival; in (**c**) predicted sequence follows the actual survival with a few exceptions; (**d**) predicted sequence follows the actual survival with high fluctuation. (**e**) Intelligible longitudinal survival curve of a patient.

However, for a few cases (e.g. Fig. 4c and d), the patient-level graphical summary shows a modarate and high fluctuation of predicted probabilistic survival during a short-interval of the temporal sequence. In such cases, understanding the basis of model predictions could be very helpful to the clinician, and we created an interactive graphical interface to allow the clinicians/end-users to infer a point-wise descriptive analysis. Clicking on a time point, the system will retrieve two key types of information - (i) visit type: by linking the note id to the structured EMR data; (ii) core findings of the visit: by matching the controlled-terms extracted from the CLEVER and Oncology dictionary, and highlighting a 5-words window around the context of the controlled-terms in the visit notes (see Fig. 4e). This intuitive illustration may help the clinician to reason on the PPES-Met prediction and perform a qualitative error-analysis. For instance, the model’s predicted survival (in green) for visit days 36–39 is relatively low and does not match with the ground truth (in blue), but, the interface shows that the low value of survival prediction is biased by the urgent hospitalization of the patient and sudden fatal conditions occurred to him. This type descriptive graphical summary can improve understanding of the model predictions, and, consequently, increase clinician acceptance.

## Discussion

### Contribution

We proposed a two-stage sequence-dependent deep learning model - Probabilistic Prognostic Estimates of Metastatic Cancer Patients (PPE-Met) which takes as input a sequence of free-text visit narratives ordered according to the visits’ timestamp, and computes as output a probability score predicting >3 months survival for each time point. The model is trained on a large dataset of 10,239 metastatic cancer patients and the performance was tested on randomly selected 1818 patients. As a ground truth label, we used categorical class labels for probabilistic prediction defined at each time point, where category 1: “Survival - positive” stands for survival up to 3months starting from the current visit date, and category 2: “Survival - negative” flagged the non-survival. The predictive accuracy of the model on test set (1818 patients) was good; AUC of the ROC curve was 0.89 (see Fig. 3). The core challenges were –

1. *how to create machine interpretable representation of relevant information present in unstructured clinical narratives* - We used a completely unsupervised novel hybrid method – Intelligent Word Embedding (IWE) that combines semantic-dictionary mapping and neural embedding technique for creating context-aware dense vector representation of unstructured free-text clinical narratives. The only required manual input for this pipeline is a list of domain-specific terms and ontology identifier.
2. *how to model the complexity of temporal data* - We designed a many-to-many RNN model using two-layer one directional stacked stateful LSTM model. The goal is to learn >3 month survival across the sequence of clinical narratives starting from the first day of visit till the end of sequence by considering the current and historic visit notes. The LSTM model allows to retain state for arbitrarily large context window. The sufficiently large training dataset helped to train the complex stacked RNN model. The model takes input a series of vectorized visit notes (created by the IWE model) ordered according to timestamp of visits, and predicts probability of survival at each time point.
3. *how to infer human interpretable explanation of prediction* – We created an interactive graphical interface that exploits the semantic mapping to extract the core findings from the visit notes and project the information in the survival curve for analysis.

### Significance

Literature^6,7^ suggests that physicians are able to predict which patients would have longer survival times, although prediction of survival was optimistic compared to actual survival by an average of 3 months. This issue can result in suboptimal treatment decisions, such as use of a two-week radiation regimen when a single day treatment would be just as effective. The main goal of the work is to improve physicians’ knowledge of their patients’ short-term prognosis to help tailor treatment intensity, improve quality of life, and reduce costs. Our proposed deep learning model, PPES-Met considers only longitudinal sequence of free-text clinical narratives (e.g. radiology reports, oncologist notes, discharge summaries), and predicts probability scores of “Survival - positive”(>3month) and “Survival - negative”(<3month) at each time point.

**Figure 5:**
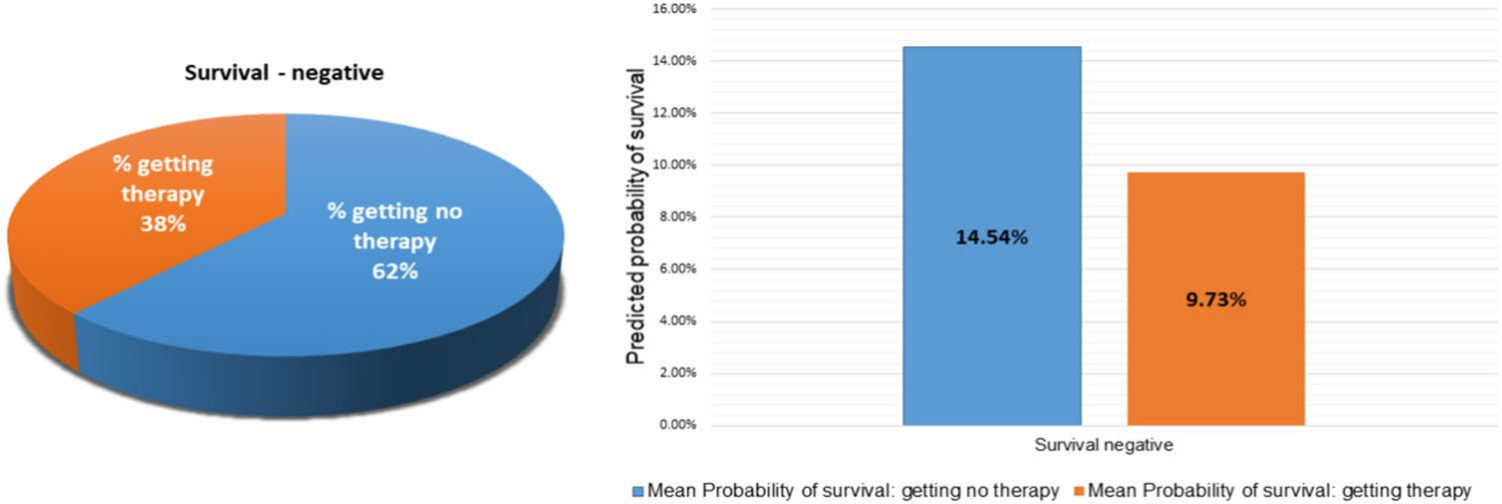
Correlating negative survival with systemic therapy data: (on left) pie chat showing 38% getting therapy in 30 days when the patients did not survived 3 months; (on right) PPES-Met prediction: bar char showing mean predicted probability of survival for those patients is less than 10%.

We tested our PPES-Met model on a combination of general group of metastatic patients and palliative radiation study and the probabilistic prediction accuracy was 0.89 AUC-ROC. The computerized model’s prediction accuracy appears superior to the physician estimates of short-term survival; thus, it can be considered as successful implementation of AI model to perform prediction. This is probably due to the capability of integrating a large amount of patient-specific facts and preserving long-term dependencies via the sequence-dependent deep learning PPES-Met model. The PPES-Met may be useful as a decision support tool in metastatic cancer patients. For instance, patients with a longer estimated life expectancy could be offered more aggressive systemic therapy regimens and longer radiation courses; patients with shorter life expectancy could be offered palliative care referral and shorter radiation courses. In order to explore potential significance, we collected the systematic therapy data (excluding oral systemic therapy prescriptions) for the patients included in the test set, and we evaluated whether or not the patient was given systematic therapy within the succeeding 30 days, and plotted against the rate of survival (see Fig. 3e). As seen from the figure, even when there is <50% chance of surviving more than 3 months, many patients are getting chemotherapy who may not benefit from it. We highlight the core findings of the plot in Fig. 5 where total 38% cases from the “Survival - negative” category receives aggressive treatment while our model predicted that the chance of survival rate is <10% for those cases. This result shows a potential benefit of our model to make informed treatment decisions in clinical scenario^29^.

### Limitations and Future Work

There are several limitations of the current work. First, a large number of patients in our current training dataset lost follow-up, since the patients often get care at several different centers around area and seen in our medical center for emergency care. This may create an inaccurate assumption about survival during the model training. However, we consulted the central cancer registry to validate the survival. Second, the time points are not equally spaced, and a single day may contribute multiple data points. This may affect the temporal dependency and introduce fluctuation in the survival curve (as seen Fig. 4). An NLP technique is needed to combine the visit data from the same day for date-based survival analysis. Third, the model was trained using single institutional data that contains biases regrading syntactic style of clinical narratives, patient populations, treatment planing. In future, we plan to include multi-institutional test dataset to validate our model. Finally, the longitudinal survival cureve for identifying the core finding of the visit notes via semantic data mapping and context analysis is not statistically evaluated in this study, but only presented as a tool for understanding basis of model prediction. In future study, we plan to evaluate the utility of our visualization approach by conducting independent sessions with radiation oncologists and analyzing their confidence in the prediction provided by with or without our visualization method.

## Methods

### Intelligent Word Embedding (IWE)

The visit notes are composed of unstructured free-text, and our strategy is to convert them into a computer manageable representation while preserving the semantic content of the narratives. For this we adopted a completely unsupervised hybrid method – an updated version of Intelligent Word Embedding (IWE) method^30,31^ that combines semantic-dictionary mapping and neural embedding technique for creating a context-aware dense vector representation of free-text clinical narratives. The method leverages the benefits of unsupervised learning along with expert-knowledge to tackle the major challenges of information extraction from clinical texts, which include uncertainty of natural language, lexical variations, use of ungrammatical and telegraphic phases, arbitrary ordering of words, and frequent appearance of abbreviations and acronyms. In the updated method, instead of averaging vector representations of every word present in the clinical notes, we only analyze the context around the controlled terms which are defined by the domain-specific dictionary since clinical notes are often lengthy and only a portion of it can be significant for survival analysis.

**Figure 6:**
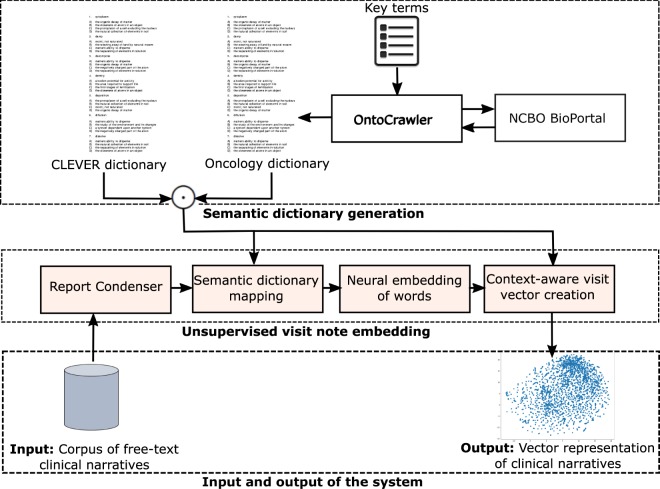
Intelligent word embedding (IWE).

Figure 6 presents the high-level model schema of IWE, which is composed of the following components:

- Report Condenser - As the majority of clinical note data are highly unstructured and noisy, we wish to clean the text data and enhance the semantic quality of the notes for achieving better insights in the later phase. We designed Report Condenser - a Python based domain-independent parser, that integrates a series of NLP pre-processing steps (stop words removal, stemming, number to string conversion) for cleaning text-data to focus on the significant concepts in the free-text narratives. Word-pairs are formed to preserve the local dependencies based on Pointwise Mutual Information. The bigrams with fewer than 50 occurrences are discarded and the top 1000 bigram collocations are concatenated into a single word. We also compute the time gap between the visit date and all the dates mentioned in the texts and convert them accordingly, e.g. 3 months ago, 1 year ago, etc.
- Semantic dictionary mapping – The semantic-dictionary mapping step uses a lexical scanner that recognizes corpus terms that share a common root or stem with a pre-defined terminology, and maps them to controlled terms to reduce term ambiguity in the clinical notes and to create more semantically structured texts. For creating the domain-specific dictionary, we applied a two-stage process.

1. OntoCrawler is a remote SPARQL query engine developed by us which, given a limited list of domain-specific key-terms and identifiers of domain-specific bio-portal ontologies, extracts all the relevant concepts (and their subclass and synonyms) using bio-portal SPARQL endpoint, resolves co-references by measuring statistical similarity, and creates a dictionary of the targeted domain. In this study, we mainly queried two terminologies - (i) National Cancer Institute Thesaurus – a vocabulary with good coverage in cancer related drug coverage and chemotherapy regimens, (ii) SNOMED Clinical Finding – with coverage in clinical abnormalities/findings. The OntoCrawler-created dictionary - Oncology dictionary, was also manually reviewed by an experienced Oncologist.
2. CLEVER^32^ is a publicly available dictionary that models the mapping of common analogies/synonyms of the terms that are frequently used in the clinical narratives and normalizes them using the formal terms derived from the terminology. For instance, {‘mother’, ‘brother’, ‘wife’, …} → FAMILY, {‘no’, ‘absent’, ‘adequate to rule her out’, …} → NEGEX, {‘suspicion’, ‘probable’, ‘possible’, …} → RISK, {‘increase’, ‘invasive’, ‘diffuse’, …} → QUAL.

We merged the two dictionaries – CLEVER and Oncology dictionary, to prepare a comprehensive terminology list (106623 controlled-terms on total), and we exploit it to recognize and map corpus terms to the controlled terms.

- Neural embedding of words – The pre-processed visit notes belonging to the training corpus were used to train the word2vec model^20^ for learning the vector embeddings of the words in a completely unsupervised manner. The word2vec adopts distributional semantics to learn dense vector representations of all words in the pre-processed corpus by analyzing the context of terms. The semantic dictionary mapping step not only considerably reduces the size of our vocabulary (40%) by mapping the words in corpus to the key terms, but also decreases the probability of out-of-the-vocabulary word encounters. Therefore, it facilitates the application of word2vec directly to parse the highly ambiguous corpus of clinical narratives. For the word2vec training, we used the skip-gram model with vector length 700 and a window width of 30, and default settings for all other parameters where the vector length and window width are optimized using grid search on the validation set. No vectors were built for terms occurring fewer than 5 times in the corpus. The trained word2vec model was used to generate the vector embedding of the words present in the pre-processed test corpus.
- Context-aware note vector creation – The context-aware visit note vectors were created by analyzing the 15 words span context-window (CWindow) of the 106,623 controlled-terms (CTerms) defined by the CLEVER and Oncology dictionary. We searched the key-terms in each report and, if a match has been found, we defined its context as the term and its surrounding 15 words. The context’s vector was then computed as mean of the context word vectors created through the trained word2vec model. Each visit vector was computed as: ${v}_{note}=\frac{1}{||N||}\,{\sum }_{c\in CTerms}(\frac{1}{||n||}\,{\sum }_{w\in CWindow}\,{v}_{w})$, where *v*_*note*_ is the visit note vector, *v*_*w*_ refers to the vector of word *w* inferred from the word2vec model, *n* is the size of context-window size (i.e. 15 in the current study), and *N* is the number of controlled-terms (CTerms) present in the report.

We also experimented with 2-step Doc2vec model^24^ that, first, modifies the word2vec algorithm to unsupervised learning of continuous representations for larger blocks of text, and then we retrain the model with lower learning rate (10 times smaller than original learning rate) on a smaller subset of labeled data. But the initial experiments showed that accuracy of the unsupervised context-aware approach for the targeted learning task performed better than the 2-step semi-supervised Doc2Vec approach.

### Design of temporal prediction model

To process the visit note embeddings in a way that accounts for longitudinal changes in the patient state, we designed a many-to-many RNN model using two-layer one-directional stacked stateful Long short-term memory (LSTM) units^33^ for learning 3 month survival across the sequence of clinical narratives. The model takes as input a series of vectorized visit notes ordered according to timestamp of visits, and predicts probability of survival 3 months after each patient visit. The choice of LSTM is based on the fact that LSTM is relatively insensitive to gap length compared to alternatives such as RNNs and hidden Markov models. The long term memory allows slow weight updates during training and encodes general information about the whole temporal visit sequence, while short-term memory has ephemeral activation and passes immediate state between successive nodes for resetting itself if a fatal condition is encountered. The LSTM includes memory about prior time points (patient visits) and thus accounts for longitudinal changes in the patient data.

For each patient (with id *i*), the sequence of clinical narratives is modeled as a series: ${X}_{i}=\,({x}_{i}^{(1)},\,{x}_{i}^{(2)},\,\ldots \mathrm{.,}\,{x}_{i}^{(n)})$, where each data point ${x}_{i}^{(t)}\in {R}^{D}$ is a real-valued vector representation of the free-text clinical narratives computed by the IWE model (see Section 3.2.1). Similarly, the targeted survival sequence is modeled as: ${Y}_{i}=({y}_{i}^{(1)},{y}_{i}^{(2)},\,\ldots \mathrm{.,}\,{y}_{i}^{(n)})$, where ${y}_{t}^{(t)}$ is a categorical variable that represents whether the patient survived more than 3 months from the *t* timepoint. Single directional LSTM units are modeled to handle the sequence-dependent vectorized visit notes and predict a probability for each time point, following the principle that at the timepoint *t* the model does not have access to the future information ${x}_{i}^{(t+1)}$ but can access the current and all the historic time points: $({x}_{i}^{(1)},{x}_{i}^{(2)},\,\ldots \,\ldots ,\,{x}_{i}^{(t-1)},{x}_{i}^{(t)})$.

In the stacked RNN layers (see Fig. 1), the first layer’s one directional LSTM block receives the input *x*^(*t*)^ and previous hidden state *h*^(*t*−1)^ and passes the current hidden state *h*^(*t*)^ to the successive LSTM blocks. The first layer’s block also passes the hidden state and current H-dimensional cell state ${c}^{(t)}\in {R}^{H}$ to the corresponding block in the upper layer. The second layer units are modeled to maintain the recurrent connections in multiple dimensions. The output estimate would be vector of probabilities across three different labels: $L=\{Survival-positive,$ $Survival-negative,Paddeddata\}$ and it is modeled as: ${\hat{y}}^{(t)}=softmax(L\circ {h}^{(t)})$, where ${\hat{y}}^{(t)}$ is the predicted survival at time *t* and *h*^(*t*)^ the hidden state of the second level. The three trainable parameters of each LSTM block are – (i) input-to-hidden weight matrix: ${W}_{x}\in {R}^{4H\times D}$, (ii) hidden-to-hidden weight matrix: ${W}_{h}\in {R}^{4H\times H}$, and (iii) bias vector: $b\,\in {R}^{4H}$.

During the training phase, our model takes as input a set of vector series that represents the sequence of the visit notes for all the patients present in training set (see Experimental setup), and optimizes the time distributed weighted cross entropy loss function: $l(Y,\hat{Y})=-\,\frac{1}{n}{\sum }_{t=1}^{n}({y}^{(t)}\,{\rm{l}}{\rm{n}}\,{\hat{y}}^{(t)}+(1-{y}^{(t)})\,{\rm{l}}{\rm{n}}\,(1-\,{\hat{y}}^{(t)})).$ *λ*^(*t*)^, where *y*^(*t*)^ actual reference survival at *t* th time point in the sequence, ${\hat{y}}^{(t)}$ represent the output of the neural network given the current sequence inputs: ${x}^{(1)},{x}^{(2)},\,\ldots .,\,{x}^{(t-1)},{x}^{(t)}$, and *λ*^(*t*)^ corresponds to the pre-defined weights.

**Figure 7:**
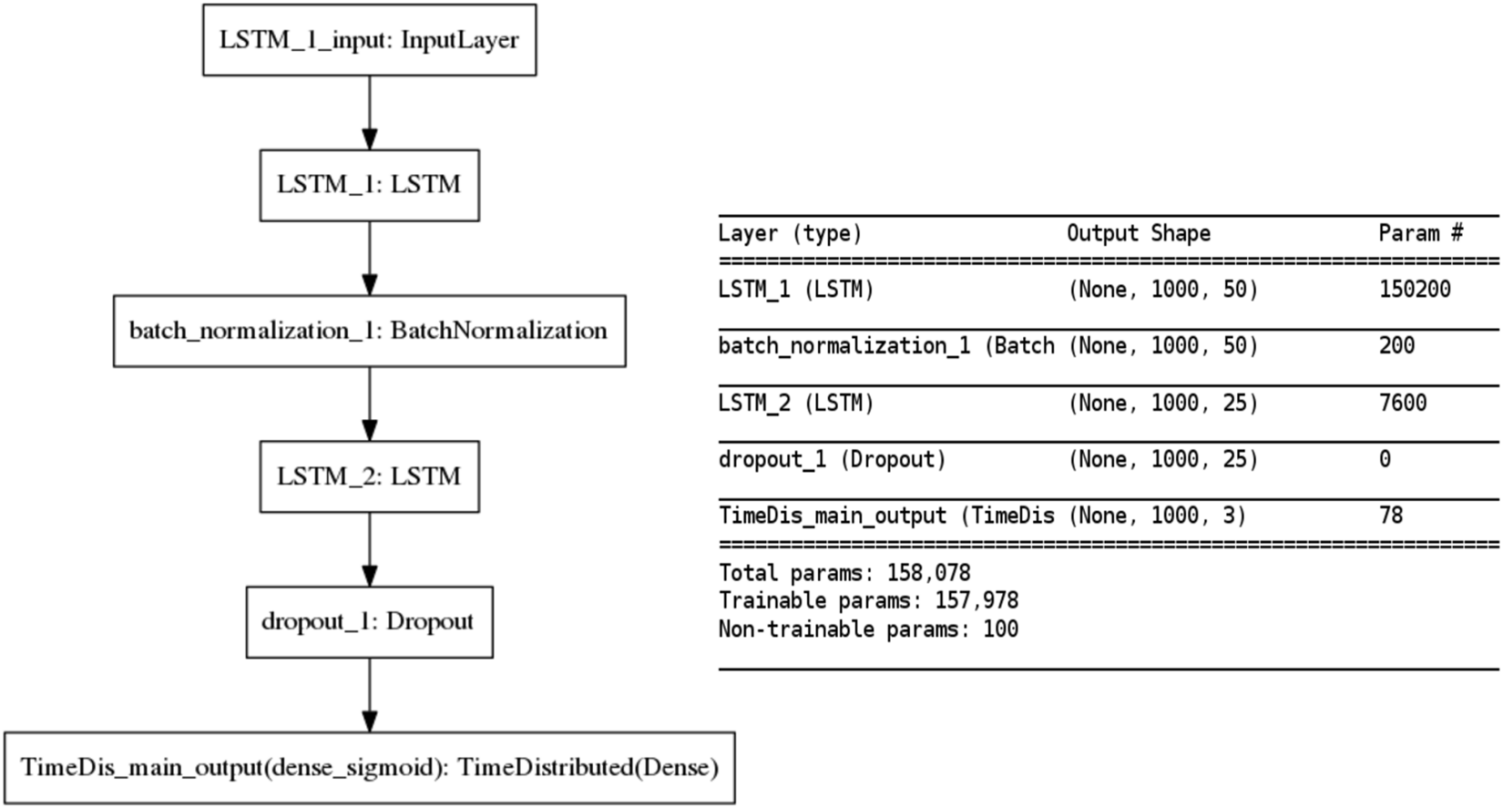
Configuration of the RNN model: unfolded configuration of the network (on left) and a summary of trainable parameters (on right).

We present the unfolded configuration of the RNN model in Fig. 1 and folded configuration in Fig. 7 (on left). We modeled layer 1 LSTM bolck with 50 hidden neurons and layer 2 block with 25 neurons where the selection is a tradeoff between the input data dimension and memory requirement for training. We used a frame-wise batch normalization layer (batch_normalization_1) between two stacked LSTM units (named as LSTM_1 and LSTM_2) and a 10% dropout layer (dropout_1) between LSTM_2 and the final predication layer (TimeDis_main_output). Batch normalization and dropout were mainly applied to achieve a faster learning and higher overall accuracy. The final TimeDis_main_output layer calculates the cross entropy loss function distributed on all time-step output where we assigned 2x weight to the “Survival - positive” class to reduce the False Negative rate. Optimization is carried out by batch Adam optimizer^34^, which iterates through subsets of the training patients and updates the model coefficients to minimize the cross entropy loss function calculated over the whole visit sequence. We particularly choose the Adam optimization scheme for handling sparse gradients problems. In Fig. 7 (on right), we also present a summary of the number of trainable parameters of the model.

## Electronic supplementary material

Supplementary Information
