# Supplementary material for: Probabilistic Prognostic Estimates of Survival in Metastatic Cancer Patients (PPES-Met) Utilizing Free-Text Clinical Narratives
Source: Sci Rep. 2018 Jul 3;8:10037. doi: 10.1038/s41598-018-27946-5 (PMC6030075; doi:10.1038/s41598-018-27946-5)
Supplement: Supplementary file 1 — Supplementary Information [file 41598_2018_27946_MOESM1_ESM.pdf]

Probabilistic Prognostic Estimates of Survival in Metastatic Cancer Patients (PPES-Met) Utilizing Free-Text Clinical Narratives

Imon Banerjee[1,*], Michael Francis Gensheimer[2], Douglas J. Wood[1], Solomon Henry[1], Sonya Aggarwal[2], Daniel T. Chang[2], Daniel L. Rubin[1]

[1]Department of Biomedical Data Science, Stanford University, Stanford, CA, USA
[2]Department of Radiation Oncology, Stanford University, Stanford, CA, USA
*imonb@stanford.edu

5-fold Cross-validation

In order to ensure generalizability of the proposed PPES-Met model, we performed 5-fold cross-validation on the training set (10,293 patients) in addition to the evaluation on the hold-out test set as described in article. We computed the mean and confidence range for all three performance evaluation metrics – AUC-ROC, AUC-PR and Brier score. A patient-level separation was used to select the test set (2048 patients) in each fold, since the notes belonging to the same patient may have significant correlation to influence the test performance. **Supplementary Figure S1.** shows the pictorial representation of our 10-fold cross-validation strategy.

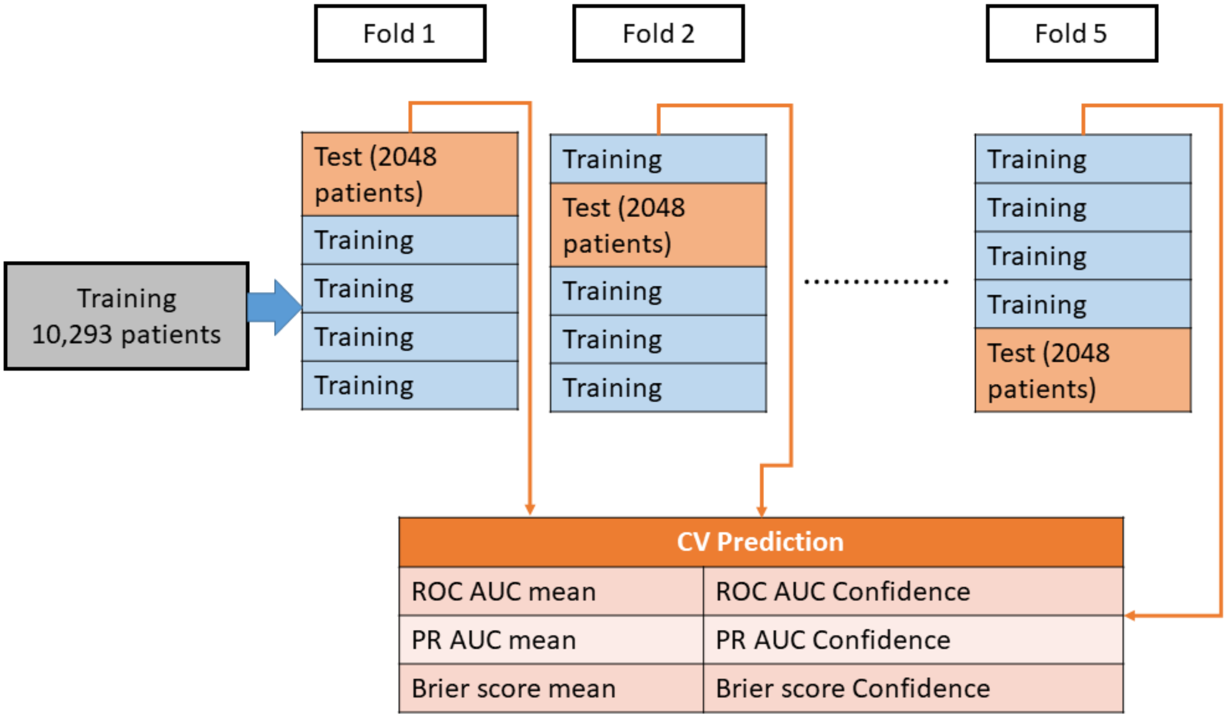

Supplementary Figure S1. 5-fold cross-validation strategy adopted in the analysis

In **Supplementary Figure S2.**, we present the mean and confidence range for the three-evaluation metrics computed over the 5-folds validation. The ROC-AUC score was 0.94+/-0.05, PR-AUC was 0.98+/-0.01, and Brier score was 0.068 +/- 0.037 which shows that the model is performing equally well for different folds. For each independent cross-fold, the learnt weights of the model were reset before training.

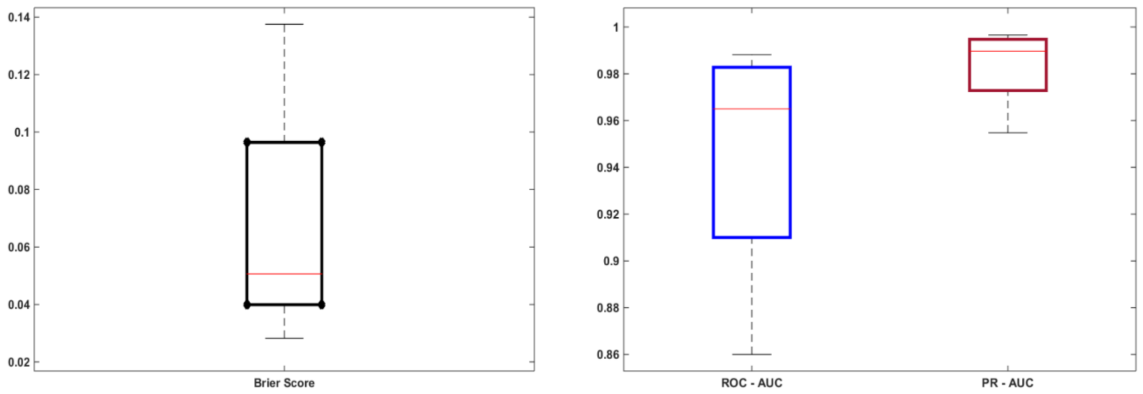

Supplementary Figure S2. 5-fold cross-validation results in terms of Brier score, ROC-AUC and PR-AUC

Ensuring no data leakage

We ensured that there is no data leakage in single-directional LSTM unit by performing patient-level tests using random vectors for 30% of the sequence data instead of original vectorized visits. We replace the original visit vectors with random vectors at the end of the series. As seen from the **Supplementary Table S3**, the *PPES-Met* model was able to differentiate between random vectors and original visits even though the numerical range of the vector is same, and survival probability drops rapidly for the time points with polluted data in the sequence.

Supplementary Table S4. Patient-level prognosis estimate with 30% polluted data

<table>
<tr><th colspan="2">Prediction test with 30% polluted visit note at the end of the series</th></tr>
<tr><td>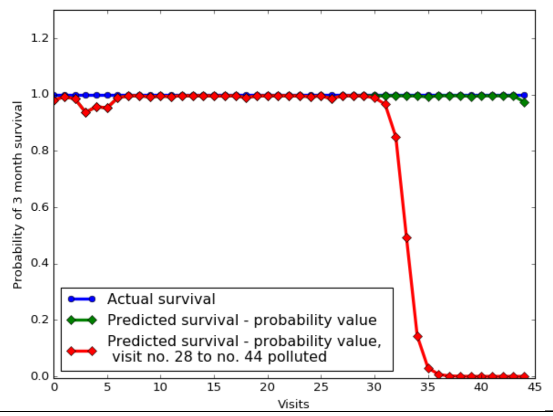
</td>
<td>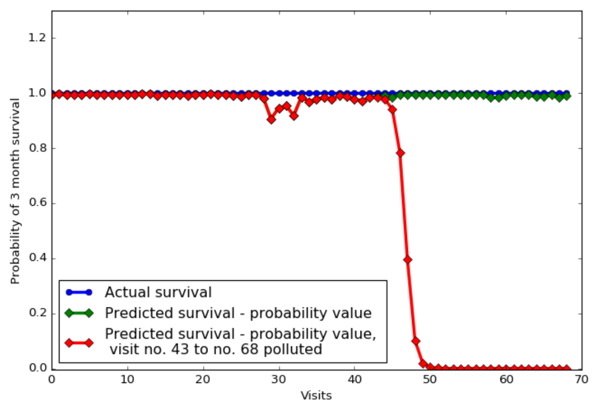
</td></tr>
</table>

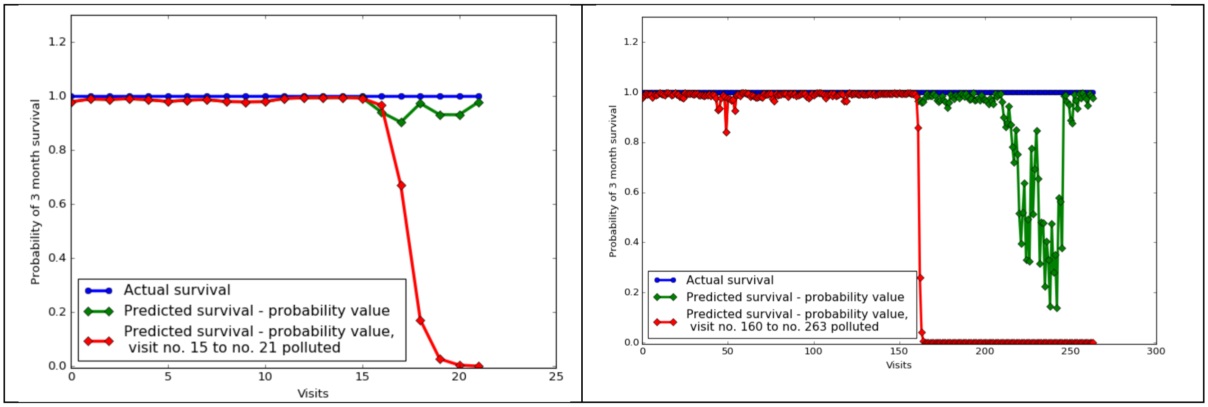

Ensuring number of visits is not the influencing factor for prediction of survival

In order to show that the survival prediction of PPES-Met model does not explicitly depend on the number of previous visits, we added a scatter plot in **Supplementary Figure S3** and computed the Pearson correlation coefficient of the predicted probability against number of previous visits. Relatively random patterns in the scatter plot and low correlation coefficient value (- 0.14) shows that there is only a minimal correlation. The low correlation represents the fact that the model is truly considering the semantic content of the visit for prediction rather than just counts of pervious encounters.

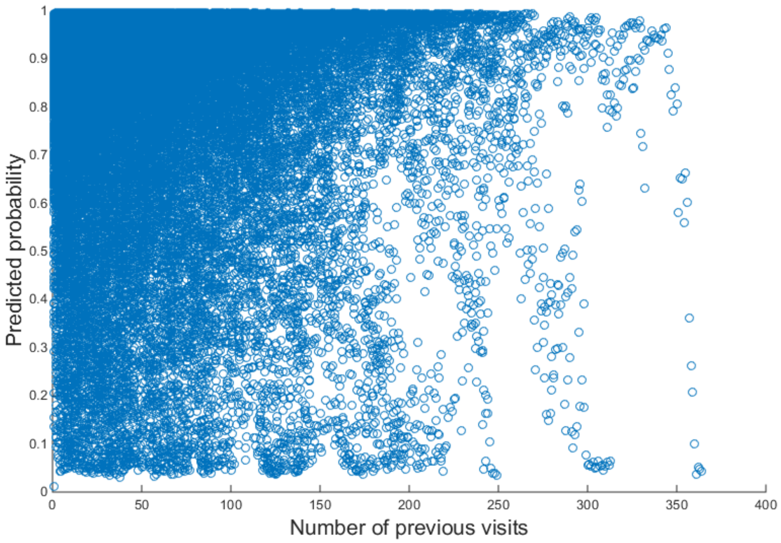

Supplementary Figure S3. Correlation plot - x-axis represents the number of previous visits and y-axis represents the predicted probability. Pearson correlation coefficient value -0.14.

Distribution of note types, categorized based on primary sites

In **Supplementary Figure S4.**, we present the distribution of note types for each primary site stratified at the patient-level. Some infrequently used note types were not represented in the test set, so the chart only shows note types that were included in the test set. As seen, presence of oncology notes is often highest, and afterwards distribution varies according to primary site. For instance, OPERATIVE REPORT is 2nd

highest for Genitourinary site, RADIATION ONCOLOGY note is 2nd highest for head and neck. But, only exception is that the GYNECOLOGY note type is highest for gynecologic site. The note distribution shows that despite the variation of note types in the test dataset, our model was able to predict the short-term survival.

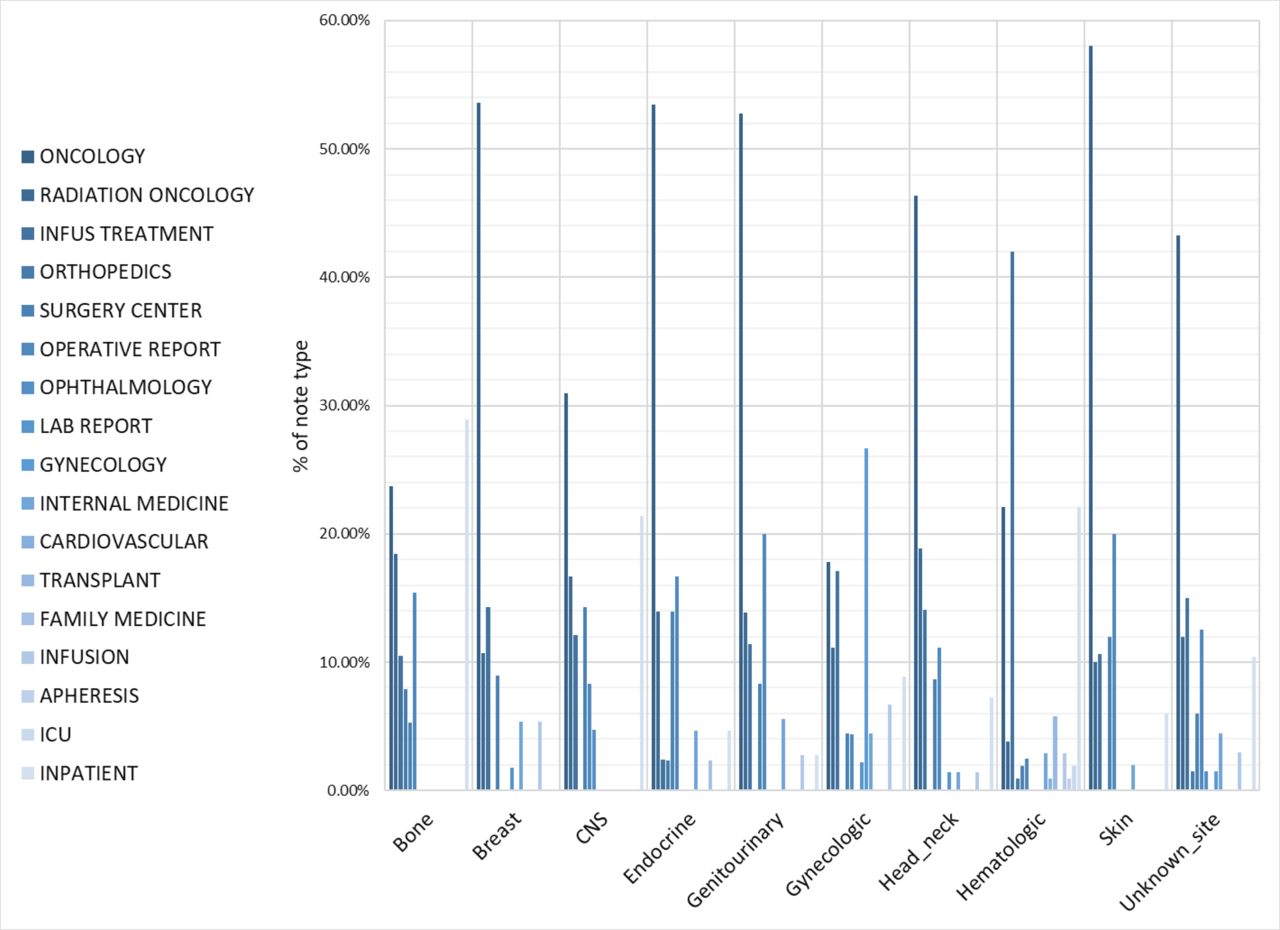

Supplementary Figure S4. Note type distribution on the test data (1,818 patients) categorized based on primary site.
